# Unraveling Huntington’s Disease: A Report on Genetic Testing, Clinical Presentation, and Disease Progression

**DOI:** 10.7759/cureus.43377

**Published:** 2023-08-12

**Authors:** Moutushi Ahmed, Debasish Mridha

**Affiliations:** 1 Internal Medicine, Khulna Medical College, Khulna, BGD; 2 Neurology, Michigan Advanced Neurology Center, Saginaw, USA

**Keywords:** disease progression, cognitive symptoms, motor symptoms, cag repeats, clinical presentation, huntington's disease

## Abstract

This study presents the clinical features and disease progression of a 39-year-old male patient diagnosed with Huntington's disease (HD). The diagnosis was confirmed by direct genetic testing, using DNA obtained from a blood sample that revealed expanded cytosine-adenine-guanine (CAG) repeats in the huntingtin gene (HD gene). The patient exhibited motor symptoms, including chorea, muscle rigidity, coordination difficulties, and speech and swallowing impairments. Cognitive symptoms comprised impaired judgment, planning difficulties, slowed thinking, memory lapses, and attention problems. The patient's progressive deterioration resulted in wheelchair dependency and increased reliance on supportive care. This report highlights the significance of genetic testing in confirming HD diagnosis and emphasizes the need for a multidisciplinary approach to manage the symptoms and improve the patient's quality of life.

## Introduction

Huntington's disease (HD) is a neurodegenerative disorder characterized by motor, cognitive, and psychiatric symptoms [1]. It arises from an abnormal amplification of CAG trinucleotide repeats in the huntingtin gene. This genetic anomaly gives rise to mutant huntingtin protein, instigating a gradual breakdown of various areas within the brain [2]. Huntington's disease (HD) is a severe condition that causes significant challenges for patients and their families. It requires comprehensive and compassionate care to manage the various symptoms and functional limitations caused by the disease.

HD typically becomes symptomatic in mid-adulthood; however, in some cases, the disease can manifest much earlier in life, known as Juvenile Huntington's disease (JHD). JHD refers to the onset of symptoms before the age of 20 years and accounts for approximately 5-10% of all HD cases. Juvenile HD presents unique challenges compared to the adult-onset form of the disease, with a more aggressive and rapidly progressive disease course. Children with JHD may experience a more severe decline in motor and cognitive functions, leading to profound disability and increased reliance on supportive care [3].

The clinical presentation of HD includes a wide range of motor symptoms, such as involuntary jerky movements, muscle stiffness, and walking difficulties, which significantly impact daily activities and mobility. Additionally, cognitive impairments, including forgetfulness, trouble concentrating, challenges in planning and organizing, and difficulties in decision-making, ultimately add to the burden of the disease. Psychiatric symptoms, such as mood swings, restlessness, irritability, apathy, lack of interest, impulsivity, and lack of emotion, further contribute to the complexity of managing HD [4-6].

This study aimed to describe HD’s clinical presentation and disease progression in a 39-year-old male patient diagnosed with confirmed expanded CAG repeats in the huntingtin gene through genetic testing.

## Case presentation

This study involves a 39-year-old married male who was diagnosed with Huntington's disease (HD) in his late 20s after undergoing genetic testing. The patient has a significant family history of HD, as both his father and sister tragically lost their lives to this devastating condition. His father succumbed to HD, while his sister experienced Juvenile HD and passed away. These familial occurrences highlight the genetic nature of HD and underscore the crucial role of genetic testing in confirming the diagnosis.

The patient married at the age of 25 years, had no children, and worked as an accountant. As time passed, he developed symptoms that affected his concentration and experienced abnormal, uncontrollable movements. His coordination deteriorated, and irritability became a constant struggle. Upon undergoing genetic testing, the diagnosis was confirmed, and his condition continued to worsen. Regrettably, the progression of the disease forced him to give up his job, as its impact on his daily life became too significant to manage. As the disease progressed, the patient's motor symptoms worsened, leading to severe disability, complete wheelchair dependency, and eventual loss of speech.

The patient's neurological examination revealed prominent chorea, generalized rigidity, and dystonia, confirming the motor manifestations of HD. Alongside these motor symptoms, significant cognitive impairments were evident, including attention difficulties, memory lapses, and executive function deficits. Behavioral changes, such as irritability, apathy, and impulsivity, further complicated the disease presentation. Due to the progressive nature of HD, the patient requires constant monitoring and assistance from two caregivers, and he relies on a liquid nutritional supplement (Ensure) with a daily intake of eight servings. The patient has been taking antiepileptic medications, valproic acid (250 mg/5 mL twice daily) and levetiracetam (500 mg twice daily), but the seizures persist, necessitating ongoing management. In addition, he has been taking Austedo (12 mg twice daily), Seroquel (50 mg thrice daily), and Ativan (1 mg) as needed. He has been seeing a psychiatrist, as regular psychiatric follow-up is crucial to address the associated mental health challenges. The patient also undergoes physical therapy (PT) and occupational therapy (OT) thrice a week to manage functional limitations. The family, especially the patient's mother and wife, experiences distress witnessing the continual decline in his health. This study underscores the intricate nature of comprehensive HD management, highlighting the importance of a multidisciplinary approach in managing diverse symptoms and enhancing the patient's overall well-being.

## Discussion

Depending on the patient's family history and any associated signs or symptoms, there are different types of tests used to detect HD, including prenatal, pre-symptomatic, and confirmatory. The diagnosis of HD in this patient was confirmed through confirmatory genetic testing, specifically the detection of expanded CAG repeats in the huntingtin gene.

HD is an autosomal dominant neurodegenerative disorder caused by abnormal expansion of CAG trinucleotide repeats in the huntingtin gene on chromosome 4p16.3. The expanded CAG repeats result in the production of mutant huntingtin protein, leading to progressive neurodegeneration in various regions of the brain.

**Figure 1:**
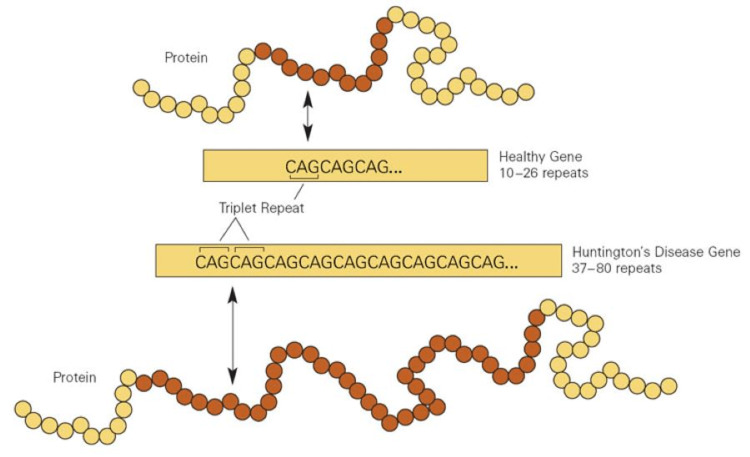
Comparison between normal gene sequence (top) and the excessive repetitions of the cytosine-adenine-guanine (CAG) nucleotide sequence in a gene from a Huntington's disease patient (bottom). The image is adapted from New NIST SRM helps improve diagnosis of Huntington's disease (April 12, 2011) (https://shorturl.at/gIZ23; public domain).

Genetic testing for HD plays a crucial role in identifying at-risk individuals, confirming the diagnosis, and providing genetic counseling. The most common method for genetic testing involves polymerase chain reaction (PCR) amplification of the CAG repeat region in the huntingtin gene, followed by fragment analysis or DNA sequencing to determine the number of CAG repeats. In individuals with HD, the CAG repeat expansion typically exceeds 36 repeats, whereas a normal allele generally has 10 to 35 CAG repeats (Figure 1).

The number of CAG repeats in the huntingtin gene can provide valuable information about disease prognosis and age of onset. There is an inverse correlation between the number of CAG repeats and the age at which symptoms first appear, known as the age of onset. Individuals with a higher number of CAG repeats tend to experience an earlier onset of symptoms. However, it is important to note that there can be considerable variability in the age of onset, even among individuals with a similar number of CAG repeats [7].

## Conclusions

This study provides crucial insights into the clinical features and progression of Huntington's disease (HD) in a patient diagnosed through genetic testing. It emphasizes the pivotal role of genetic testing in confirming HD diagnosis, especially for individuals with a family history of the disease. Early detection is highlighted, underscoring the importance of timely intervention and comprehensive symptom management to enhance the overall quality of life for affected individuals. An interdisciplinary approach involving specialists from neurology, psychiatry, physical therapy, and occupational therapy is essential in addressing the diverse manifestations of this debilitating neurodegenerative disorder. Through collaboration, healthcare professionals can optimize symptom control, provide support for the patient's well-being, and offer understanding and assistance to families facing the challenges of HD. Ultimately, this collaborative approach aimed to improve HD management and the quality of life for patients and their loved ones, offering hope and compassion throughout the journey of dealing with HD.
